# A Computational Model to Predict the Causal miRNAs for Diseases

**DOI:** 10.3389/fgene.2019.00935

**Published:** 2019-10-03

**Authors:** Yuanxu Gao, Kaiwen Jia, Jiangcheng Shi, Yuan Zhou, Qinghua Cui

**Affiliations:** ^1^Department of Biomedical Informatics, Department of Physiology and Pathophysiology, Center for Noncoding RNA Medicine, MOE Key Lab of Cardiovascular Sciences, School of Basic Medical Sciences, Peking University, Beijing, China; ^2^Center of Bioinformatics, Key Laboratory for Neuro-Information of Ministry of Education, School of Life Science and Technology, University of Electronic Science and Technology of China, Chengdu, China

**Keywords:** miRNAs, disease, miRNA–disease association prediction, miRNA functional similarity, network biology

## Abstract

MicroRNAs (miRNAs) are one class of important noncoding RNA molecules, and their dysfunction is associated with a number of diseases. Currently, a series of databases and algorithms have been developed for dissecting human miRNA–disease associations. However, these tools only presented the associations between miRNAs and disease but did not address whether the associations are causal or not, a key biomedical issue that is critical for understanding the roles of candidate miRNAs in the mechanisms of specific diseases. Here we first manually curated causal miRNA–disease association information and updated the human miRNA disease database (HMDD) accordingly. Then we built a computational model, MDCAP (MiRNA-Disease Causal Association Predictor), to predict novel causal miRNA–disease associations. As a result, we collected 6,667 causal miRNA–disease associations between 616 miRNAs and 440 diseases, which accounts for ∼20% of the total data in HMDD. The MDCAP model achieved an area under the receiver operating characteristic (ROC) curve of 0.928 for ROC analysis by independent test and an area under the ROC curve of 0.925 for ROC analysis by 10-fold cross-validation. Finally, case studies conducted on myocardial infarction and hsa-mir-498 further suggested the biomedical significance of the predictions.

## Introduction

MicroRNAs (miRNAs) are a class of ∼22-nucleotide-long small noncoding RNA that mediate gene posttranscriptional regulation. MiRNAs could suppress gene expression by targeting the 3′ untranslated region of mRNAs (Carthew and Sontheimer, 2009; Ameres and Zamore, 2013). With the development of high-throughput sequencing technology, over 2,600 mature miRNA molecules have been identified in human body, and these miRNAs regulate more than 15,000 genes in total (Chou et al., 2018; Kozomara et al., 2019). Increasing studies reveal that miRNAs are involved in many essential biological processes such as proliferation, differentiation, apoptosis, and development (Esteller, 2011; Gebert and MacRae, 2019). And the dysfunction of miRNAs is associated with large number of diseases, including but not limited to cancers, cardiovascular diseases (CVDs), and neurological disorders (Esteller, 2011; Small and Olson, 2011; Wang et al., 2019b). Thus, databases for miRNA–disease associations are increasingly important for dissecting the roles of miRNAs in diseases. For this purpose, in 2007, we built the human miRNA disease database (HMDD) (Lu et al., 2008) and launched versions 2 and 3 in 2013 and 2018, respectively (Li et al., 2014; Huang et al., 2019). According to the latest update of HMDD, 35,547 experimentally verified miRNA–disease associations have been manually collected, which cover 1,206 miRNAs and 893 diseases.

Although these miRNA–disease association data have significantly benefited the research of the disease-related miRNAs, the experimental methods for verifying miRNA–disease associations are labor-intensive and time-consuming, and therefore computational approaches for predicting novel miRNA–disease associations are also highly warranted to facilitate diagnosis, prognosis, and treatment of complex diseases (Liu et al., 2008; Chen et al., 2019; Wang et al., 2019a; Yu et al., 2019). Indeed, a series of computational methods for predicting potential miRNA–disease associations have been developed, for example, (Jiang et al., 2010; Chen et al., 2012; Wang et al., 2019a; Yu et al., 2019). However, most of the algorithms used miRNA–disease associations in HMDD as training set, and many of them achieved a reliable performance (Chen and Huang, 2017; Chen et al., 2018a). According to a recent review, these methods can be divided into four categories: score function–based, complex network algorithm–based, machine learning–based, and multiple biological information–based models (Chen et al., 2019).

Nevertheless, in biomedical views, the miRNA–disease associations can be causal (e.g., miRNAs that could result in disease phenotypes when permutated) or passive (e.g., differentially expressed miRNAs with no significant involvement of disease mechanism). Intuitively, the causal associations are more important for better understanding of the roles of miRNAs in diseases, efficiently discovering new biomarkers for diseases progress and precisely dissecting the putative miRNA therapeutic targets for the intervention of diseases. Yet, to our knowledge, none of the current available databases and algorithms addressed the causality information between miRNAs and diseases.

Given the importance of the causality information in miRNA–disease study, recently we reviewed all associations in the latest version of HMDD (January 2019, HMDD v3.1) and identified the causal miRNA–disease associations among them. With these causal association data, we further developed a prediction model named MDCAP (MiRNA-Disease Causal Association Predictor) based on the label propagation algorithm for predicting potential causal miRNA–disease associations. Ten-fold cross-validation and independent testing were performed to evaluate the model performance, and several predictions were also confirmed by the latest experimental results.

## Materials and Methods

### Data Collection

The miRNA–disease association dataset was downloaded from the latest HMDD database (http://www.cuilab.cn/hmdd/) (Huang et al., 2019), which was updated recently (January 2019, HMDD v3.1). The miRNA family information was obtained from miRBase v22 (http://www.mirbase.org/) (Kozomara et al., 2019). And the single-nucleotide polymorphism (SNP) data were downloaded from dbSNP (ftp://ftp.ncbi.nih.gov/snp/organisms/human_9606/VCF/). Medical Subject Headings data were downloaded from its website (https://www.nlm.nih.gov/databases/download/mesh.html).

### Curation of Causal miRNA–Disease Associations

**Figure 1:**
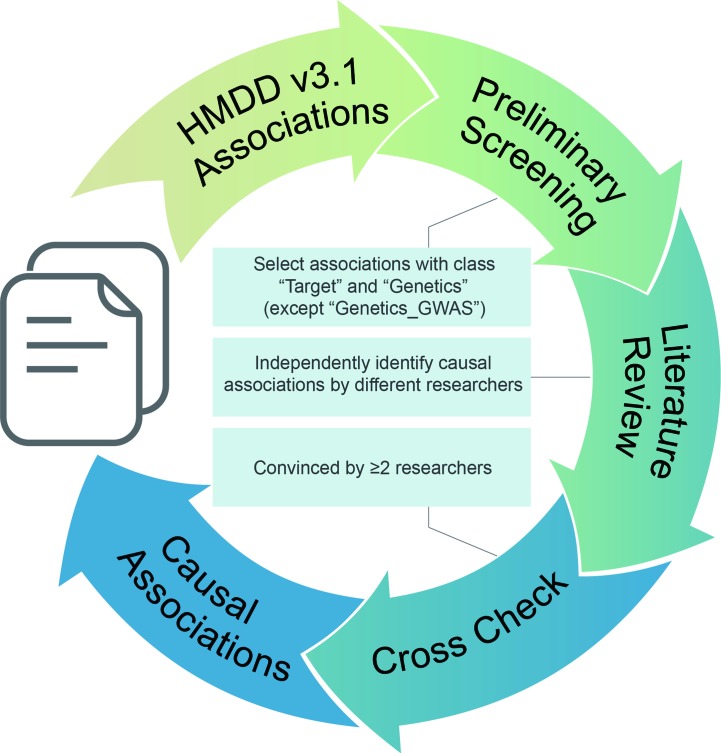
Curation workflow of the causal miRNA–disease associations.

To get reliable results, we performed the manual literature review following the workflow in **Figure 1**. First, we used the evidence code provided in HMDD v3.1 for preliminary screen. Records with the evidence code classes “Target” and “Genetics” were selected as candidate except those with “Genetics_GWAS” label for the reason that genome-wide association study presents associations between genetic loci and disease but does not present causality. Next, causal associations are identified independently by different curators according to the following criteria: (1) the corresponding study must contain gain-of-function and/or loss-of-function experiments on the given miRNAs; (2) functional experiments must be conducted in cell line and/or disease animal model; (3) those associations of which miRNAs could enhance drug effects but have no contributions to diseases are excluded. Finally, one curator’s results were double checked by another curator, and only those confirmed by at least two researchers are marked as causal miRNA–disease associations.

### Workflow of MDCAP

**Figure 2:**
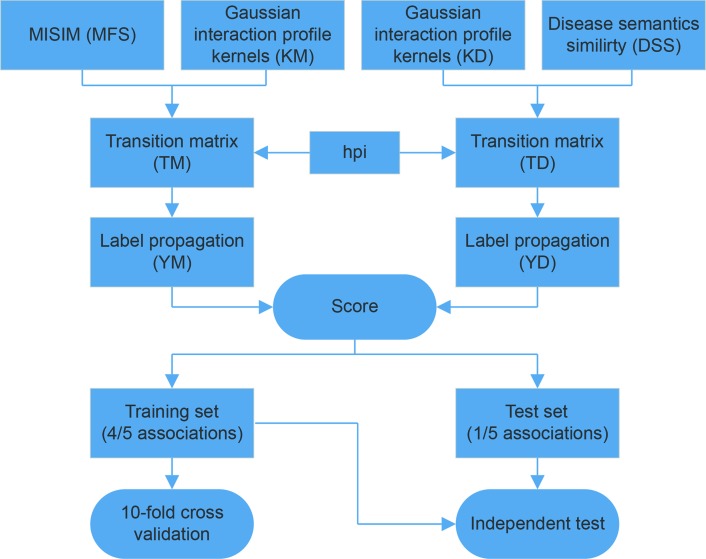
Workflow of the MDCAP prediction model.

MDCAP performs the label propagation algorithm on miRNA similarity matrix and disease similarity matrix to predict potential causal miRNA–disease associations based on known causal associations. The workflow of MDCAP is shown in **Figure 2**. First of all, we calculated the disease sematic similarity and miRNA functional similarity. In order to better using the topological information of known miRNA–disease causal association network, we also calculated the Gaussian interaction profile kernel similarity of miRNAs and diseases (van Laarhoven et al., 2011; Chen et al., 2016). As we found miRNAs with a higher number of causal diseases were more conserved, we adjusted the transition matrices with hub promoted index. Then we generated the transition matrices for label propagation by integrating similarities above for miRNAs and diseases, respectively. Finally, we performed the label propagation on two matrices separately, and the final score of each miRNA–disease pair consisted of the two label propagation results. To evaluate the performance of MDCAP, we performed independent test and 10-fold cross-validation on it. One-fifth causal associations were randomly extracted from all known causal associations as test set and rest associations were used as training set. 10-fold cross-validation was performed on training set to optimize parameters. More details are described in **File S1**. Source codes of MDCAP are available at https://github.com/cuppeanuts/MDCAP. 

## Results

### Overview of the Causal miRNA–Disease Associations

**Figure 3:**
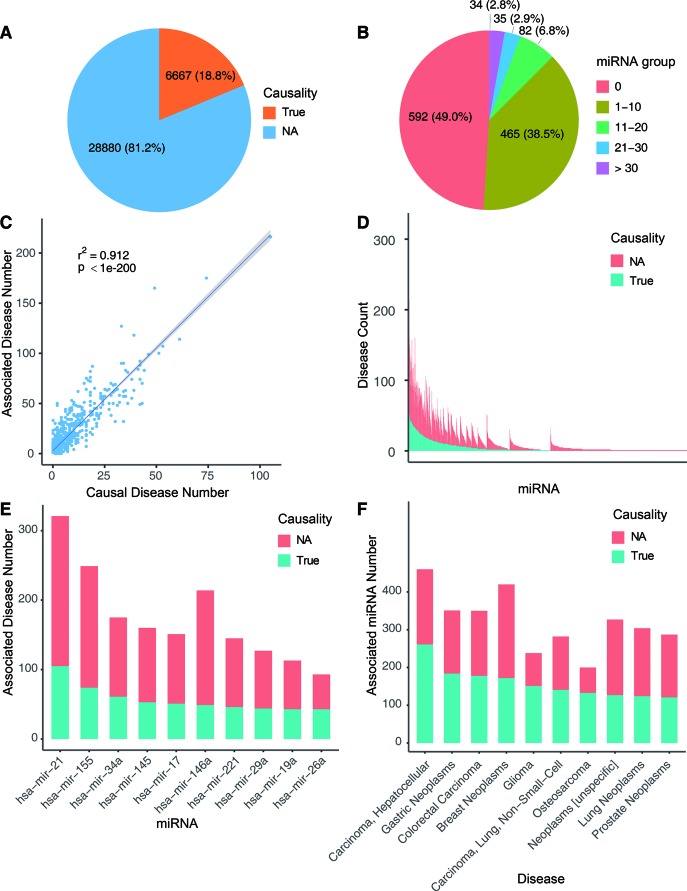
Overview of the causal miRNA–disease associations. **(A)** Pie chart showing the proportion of causal miRNA–disease associations. **(B)** Pie chart depicting the fractions of miRNAs with different causal disease numbers. **(C)** Correlation between the associated disease numbers and the causal disease numbers. Blue line shows the smooth line based on the linear model smoothing, and the shadow indicates 95% confidence interval. **(D)** Bar plot shows the associated disease numbers of all miRNAs. MiRNAs were ranked by causal disease numbers and total associated disease numbers. Blue bar represents causal disease numbers, and red bar represents noncausal disease numbers. **(E)** The top 10 miRNAs with the highest numbers of causal-associated diseases. **(F)** The top 10 diseases with the highest numbers of causal miRNAs.

Using the workflow in **Figure 1**, we curated the causal miRNA–disease association data, and using the workflow in **Figure 2**, we implemented an algorithm (MDCAP) to predict novel causal miRNA–disease associations (see details in *Materials and Methods*). As a result, there are in total 35,547 miRNA–disease associations in HMDD v3.1 including 1,206 miRNAs and 893 diseases. By manual curation, 6,667 miRNA–disease associations are labeled as causal, and these data represent about one-fifth of the total miRNA–disease associations in HMDD v3.1 (**Figure 3A**, http://www.cuilab.cn/hmdd). These miRNA–disease causal association data contain 616 miRNAs and 440 diseases. We assigned all miRNAs in HMDD v3.1 into five groups according to their causal disease numbers (cdn). We found that ∼50% of all the miRNAs have no causal information for any diseases, while ∼3% of all the miRNAs are causal in more than 30 diseases (**Figure 3B**). Moreover, the number of causal-associated diseases of one miRNA is significantly correlated with the total number of diseases that are associated with this miRNA (**Figure 3C**). However, there are also plenty of miRNAs associated with many diseases but without known causal-associated diseases (**Figure 3D**). For example, many mental disorders such as autistic disorder are associated with lots of miRNAs, but currently none of these miRNAs have been reported to be causal, suggesting that it could be more difficult to identify causal miRNAs for these diseases. The top 10 miRNAs with the highest number of causal-associated diseases are shown in **Figure 3E**. The top 10 diseases with the highest number of causal miRNAs are shown in **Figure 3F**. We found that cancers, as the well-known complex diseases, have occupied the top list.

### Correlation Between miRNA Cdn and miRNA Conservation

**Figure 4:**
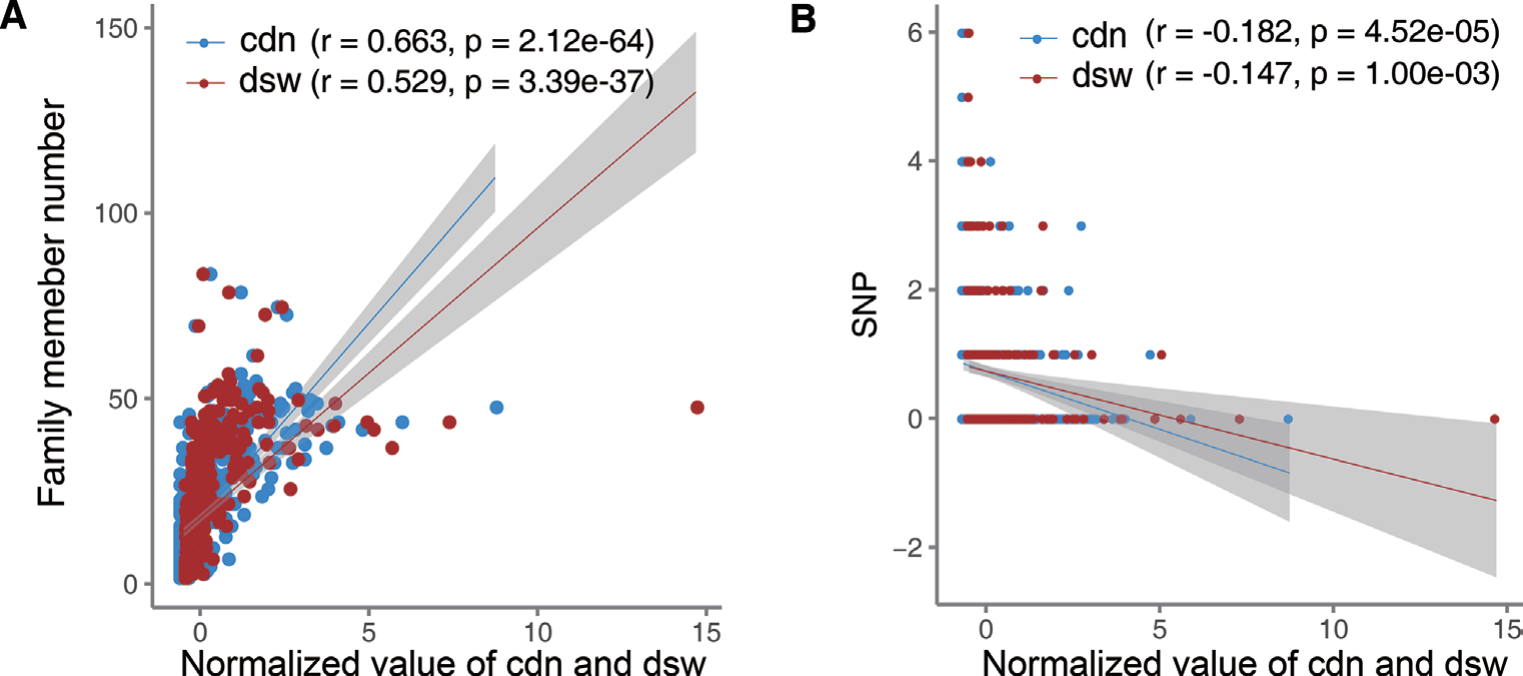
Correlation between causal disease number of miRNAs and miRNA conservation. The *X* axis represents normalized cdn or dsw. Lines show the smooth lines based on the linear model, and the shadow indicates 95% confidence interval. **(A)** Correlation with the miRNA family member number. **(B)** Correlation with the number of SNPs harbored in miRNA precursors.

Conservation of a gene could indicate the importance of this gene in organism development. Therefore, miRNAs with higher conservation may be causally associated with more diseases. Thus, we investigated the relationship between cdn of miRNAs and their conservation. Here we used the number of miRNA family members and the number of SNP sites harbored in miRNA precursors to represent miRNA conservation (**File S2**). We found that cdn of miRNAs is positively correlated with miRNA family member numbers (**Figure 4A**). And cdn also has a significant negative correlation with SNP number (**Figure 4B**). Besides, disease spectrum width (dsw) is a metric measuring the total number of associated diseases, no matter whether causal or not, for each miRNA. We also noted that cdn of miRNAs has a higher correlation with miRNA conservation than dsw in terms of both indexes (**Figure 4**), indicating that the number of causal-associated diseases could a better indicator of evolutionarily conserved (and more likely functional important) miRNAs than the total number of associated diseases.

### Prediction of Causal miRNA–Disease Associations

**Figure 5:**
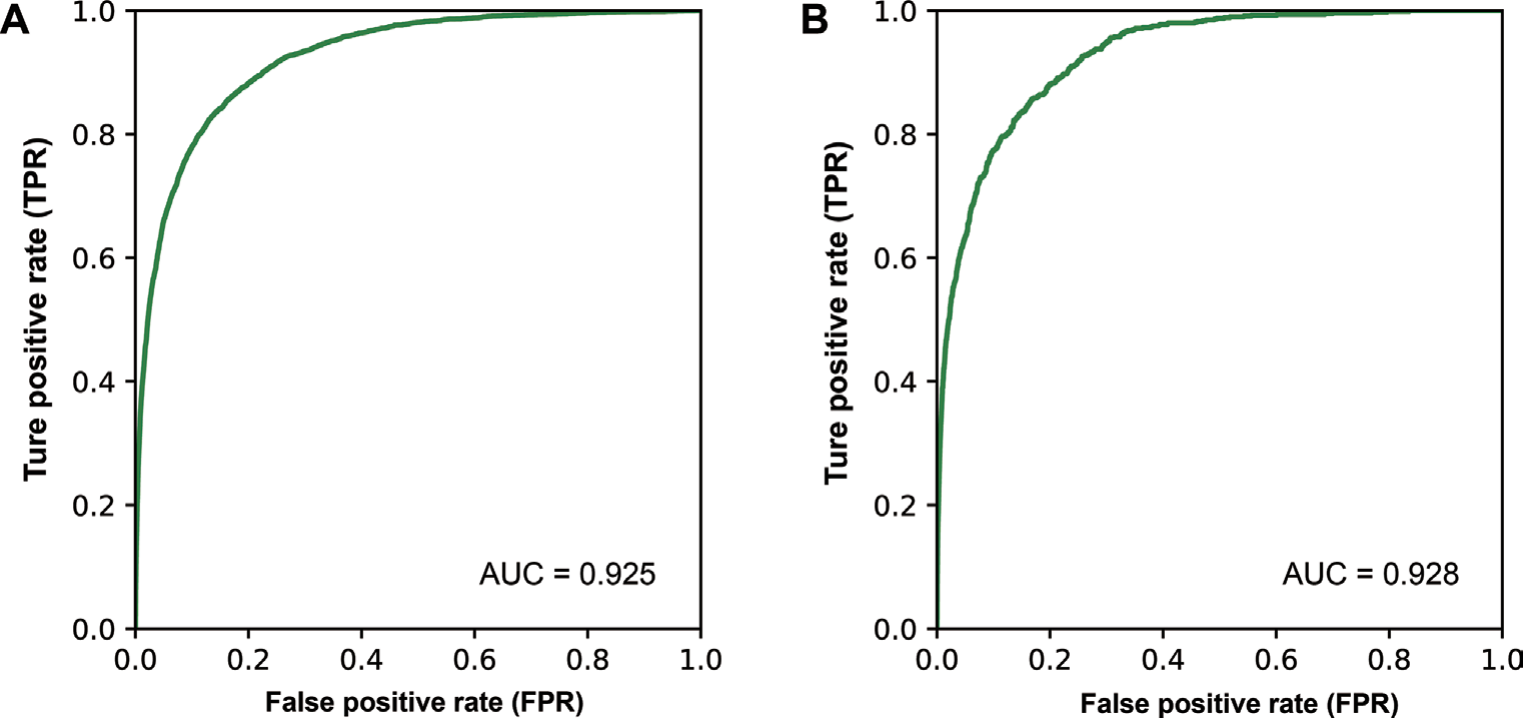
ROC curve showing the performance of MDCAP. **(A)** ROC curve of 10-fold cross-validation. **(B)** ROC curve of independent testing set.

In order to efficiently discovery new causal miRNA–disease associations, we developed a prediction model named MDCAP for predicting causal miRNA–disease associations. MDCAP was built based on label propagation algorithm. MDCAP would give a score for every miRNA–disease pair in training set. The closer the score is to 1, the more likely the miRNA is causal for the disease. To evaluate the performance of MDCAP to infer potentially causal associations between miRNAs and diseases, 20% associations were randomly extracted from all known causal associations as the independent testing set, and the rest causal associations were used as the training set. Tenfold cross-validation was implemented on training set to optimize the parameters. During each round of cross-validation, the causal associations were also randomly divided into a training set and a test set. MiRNA and disease similarity matrices were generated solely from the training set, and associations where the miRNA or disease name was not included in training set were discarded from the testing set since no prediction result will be produced for such associations. Based on the prediction scores of MDCAP on the testing samples, receiver operating characteristic (ROC) curve was plotted according to true positive rate (sensitivity) and false positive rate (1 − specificity) at different thresholds. Then the area under the ROC curve (AUC) was calculated to estimate the performance of MDCAP. As shown in **Figure 5**, MDCAP achieved overall reliable prediction accuracy with an AUC of 0.925 on 10-fold cross-validation and AUC of 0.928 on the independent testing set. Moreover, we repeated the cross-validation and independent test randomly for 10 times. As a result, the average AUCs of the independent test and cross-validation are 0.930 and 0.927, respectively. We also performed 5-fold cross-validation and independent test randomly for 10 times. The results showed that the average AUCs of the independent test and cross-validation are 0.932 and 0.922, respectively.

### Case Studies About the Prediction Results

With MDCAP scores, we can predict potential causal miRNAs for a given disease. CVD is one leading cause of deaths and disability-adjusted life-years globally, and the global number of deaths from CVD has been increasing during the past decades (Joseph et al., 2017). Myocardial infarction (MI), referred to as heart attack, is an acute coronary syndrome in which a coronary artery is blocked often by thrombus and results in heart failure. Here we used MDCAP to predict miRNAs that might play a causal role in the mechanism of MI. We found that hsa-mir-155 has a score of 0.243 and is ranked first out of all potential miRNAs (**Table 1**). And the causal association between mir-155 and MI has been verified in the latest literature that has not been recorded in HMDD v3.1 (Guo et al., 2019). In this article, researchers found that the expression of miR-155 was dynamically upregulated in murine hearts subjected to MI, and the inhibition of miR-155 expression with AMO-155 (an antisense inhibitor oligodeoxyribonucleotides against miR-155) could significantly increase cell viability, reduce cell apoptosis, and improve the cardiac function. As the authors used carefully designed experimental intervention to modulate the miRNA and the modulation has a clear effect on disease progression in disease animal model, it can be confirmed that the association between miR-155 and MI is indeed causal.

**Table 1:** The top 5 miRNAs with the highest causal potential for MI.

miRNA	Disease	Score	Rank	PMID
hsa-mir-155	Myocardial infarction	0.242872	1	31191799
hsa-mir-145	Myocardial infarction	0.190895	2	–
hsa-mir-221	Myocardial infarction	0.166031	3	–
hsa-mir-26a	Myocardial infarction	0.158790	4	–
hsa-mir-19a	Myocardial infarction	0.157822	5	–

Similarly, MDCAP could also be helpful to find new causal-associated disease of a miRNA. Hsa-mir-498 has been proven to be aberrantly expressed in several human malignancies and allergic diseases. Here we used MDCAP to predict causal disease associations of hsa-mir-498. We found that hepatocellular carcinoma obtained a score of 0.223 and is ranked the first among all potential diseases (**Table 2**). This result also has been verified in the latest literature that has not been recorded in HMDD v3.1 (Zhang et al., 2019). In this article, the authors found that miR-498 was significantly downregulated in liver cancer patient tissues, and the overexpression of miR-498 markedly inhibited liver cancer cell proliferation, migration, and invasion. Clearly, the results of this article provide a direct evidence for the causal miRNA–disease association between mir-498 and hepatocellular carcinoma, where the modulation of the miRNA has a clear effect on disease progression in disease animal model. Finally, we applied MDCAP to predict potentially causal miRNA–disease associations (in the download page of HMDD).

**Table 2:** The top 5 disease with the highest causal potential by hsa-mir-498.

miRNA	Disease	Score	Rank	PMID
hsa-mir-498	Carcinoma, hepatocellular	0.223151	1	30592286
hsa-mir-498	Stomach neoplasms	0.170807	2	–
hsa-mir-498	Colorectal neoplasms	0.143714	3	–
hsa-mir-498	Glioma	0.124206	4	–
hsa-mir-498	Neoplasms	0.121018	5	–

## Discussion

Increasing evidence shows that miRNAs are involved in many diseases such as cancers, CVDs, and neurodegenerative diseases (Esteller, 2011). However, most of them could be merely “passenger miRNAs,” which are passively altered during the progression of diseases. Identification of disease causal miRNAs is more helpful for understanding diseases. Moreover, targeting miRNAs is becoming a new strategy in drug discovery (Warner et al., 2018); the causal information could also be helpful for exploring therapeutic target miRNAs more precisely and quickly. Many algorithms have been developed to predict novel miRNA–disease associations but not address the causal information. (Chen et al., 2018b; Chen et al., 2018c) Here we curated the causal associations from the latest version of HMDD database and came up with a model MDCAP for miRNA–disease causal association prediction. MDCAP achieved a reliable performance on 10-fold cross-validation and independent testing data. Besides, several latest publications also support our prediction. These results indicated that MDCAP is a reliable model for miRNA–disease causal association prediction. With causal information, users could perform various analyses including, but not limited to, discovering therapy target miRNAs and performing functional enrichment analysis. However, there exist some limitations. One major limitation is that the prediction space for miRNAs was limited to the existed miRNAs in the miRNA matrix. In this study, the miRNA matrix was generated using the causal miRNA–disease association data, which limited the predictive model to the miRNAs included in the causal miRNA–disease association dataset. One solution is to generate the matrix based on other dataset, for example, miRNA–target interaction, expression, and sequence. Finally, we believe that these data and tools represent a useful resource for future investigations on the miRNAs’ involvement in the causal disease mechanisms.

## Data Availability Statement

Publicly available datasets were analyzed in this study. This data can be found here: http://www.cuilab.cn/hmdd. 

## Author Contributions

QC conceived the project. QC and YZ designed the experiments. YG, KJ, and JS conducted the experiments. YG analyzed the data with supervision from YZ and QC. YG, YZ, and QC wrote the manuscript. All authors read and approved the final manuscript.

## Funding

This work was supported by the grants from the National Key R&D Program (2016YFC0903000 to QC); the Natural Science Foundation of China (81670462 and 81970440 to QC, 31801099 to YZ); and the Fundamental Research Funds for Central Universities of China (BMU2017YJ004 to YZ).

## Conflict of Interest

The authors declare that the research was conducted in the absence of any commercial or financial relationships that could be construed as a potential conflict of interest.

## Supplementary Material

File S1 Supplementary text for the methods.

File S2 Number of causal diseases, disease spectrum width, number of family members, and number of SNPs for pre-miRNAs.
