# Supplementary material for: A Computational Model to Predict the Causal miRNAs for Diseases
Source: Front Genet. 2019 Oct 3;10:935. doi: 10.3389/fgene.2019.00935 (PMC6786093; doi:10.3389/fgene.2019.00935)
Supplement: File S1 — Supplementary text for the methods. [file DataSheet_1.docx]

**SUPPLEMENTARY METHODS**

**Disease semantic similarity**

MeSH database provides a systematic classification for diseases, which is useful for learning the relationship between diseases. According to MISIM algorithm [[1](#_ENREF_1)], for each disease in MeSH database, its similarity with other disease can be described as a directed acyclic graph (DAG), *DAG(A) = (A, T(A), E(A))*, where *T(A)* represents the node *A* and its ancestor nodes, *E(A)* represents the set of direct edges from a parent node *t* to a child nodes *t’*. Then the sematic value of disease *A*, *DV(A)*, could be calculated as follows:

$DV(A)=\sum_{t\in T(A)} D_{A}(t)$ (1)

where

$\left\{\begin{matrix} D_{A}(A)=1 \\ D_{A}(t)=\max\{\Delta*D_{A}(t')|t'\in children of t\} if t\neq A \end{matrix}\right.$ (2)

In above formula, Δ is the semantic contribution factor. Based on the assumption that diseases sharing large parts of their DAGs are more like to have higher semantic similarity, the sematic similarity of two diseases was calculated as

$DSS(A,B)=\frac{\sum_{t\in T(A)\cap T(B)} (D_{A}(t)+D_{\text{B}}(t))}{DV(A)+DV(B)}$ (3)

**MiRNA functional similarity**

MiRNA functional similarity score was calculated according to our previous work, which was based on the assumption that functionally similar miRNAs tend to be associated with phenotypically similar diseases [[2](#_ENREF_2)]. Briefly, assuming that *X, Y* represent two miRNA and *XD, YD* represents their causatively associated disease sets. Then miRNA functional similarity can be calculated as follows:

$MFS(X,Y)=\frac{\sum_{x\in XD} MS(x,YD)+\sum_{y\in YD} MS(y,\text{XD})}{Counts(XD)+Counts(YD)}$ (4)

where

$\left\{\begin{matrix} MS(x,YD)=\max_{y\in YD} (DSS(x,y)) \\ MS(y,XD)=\max_{x\in XD} (DSS(x,y)) \end{matrix}\right.$ (5)

**Gaussian interaction profile kernel similarity for miRNAs and diseases**

The calculation of Gaussian interaction profile kernel similarity is also based on the assumption that miRNAs with similar functions tend to be associated with similar disease and vice versa and has been detailed described in previous literatures [[3-5](#_ENREF_3)]. Briefly, we used a binary vector *d(i)* represents the interaction profile of disease *i* with every miRNA. Then the Gaussian kernel similarity of diseases could be calculated as follows:

$KD(i,j)=\exp(-\gamma_{d}||d(i)-d(j)||^{2})$ (6)

where the parameter *γ_d_* was used to control bandwidth and defined as:

$\gamma_{d}=\gamma_{d}'/(\frac{1}{n_{d}}\sum_{i=1}^{n_{d}} ||d(i)||^{2})$ (7)

That is, we normalize the new bandwidth parameter by dividing it by the average number of causal miRNAs per disease. And the new bandwidth parameter *γ_d_’* was set to 1.

Besides, Gaussian kernel similarities for miRNAs could be calculated in the similar fashion as those for diseases:

$KM(i,j)=\exp(-\gamma_{m}||m(i)-m(j)||^{2})$ (8)

$\gamma_{m}=\gamma_{m}'/(\frac{1}{n_{m}}\sum_{i=1}^{nm} ||m(i)||^{2})$ (9)

**Integrating similarity information**

The disease transition matrix of label propagation was obtained by integrating disease sematic similarity and disease Gaussian kernel similarity. Assuming that *CM(i)* represents the causal miRNA set of disease *i* and |*CM(i)*| represents the number of *CM(i)*, the transition probability between disease *i* and disease *j* could be defined as follows:

$TD(i,j)=\left\{\begin{matrix} hpi\cdot DSS(i,j), DSS(i,j)\neq0 \\ hpi\cdot KD(i,j), otherwise \end{matrix}\right.$ (10)

Where the *hpi* is the hub promote index term which was introduced for bias adjustment, the *hpi* could be defined as:

$hpi=\frac{|CM(i)\cap CM(j)|}{\min\{|CM(i)|,|CM(j)|\}}$ (11)

Similarly, the transition probability between miRNA *i* and miRNA *j* can be defined as follows:

$TM(i,j)=\left\{\begin{matrix} hpi\cdot MFS(i,j), MFS(i,j)\neq0 \\ hpi\cdot KM(i,j), otherwise \end{matrix}\right.$ (12)

where

$hpi=\frac{|CD(i)\cap CD(j)|}{\min\{|CD(i)|,|CD(j)|\}}$ (13)

**Label propagation**

Label propagation is a semi-supervised method to infer unlabelled samples based on labelled information. Generally, the label propagation process can be described as follows:

$Y_{t+1}=(1-\alpha)\cdot T\cdot Y_{t}+\alpha\cdot ADJ$ (14)

where *t* is step and *Y_t+1_* is the iteration results of label propagation after *t* steps propagation. *α* is the restart probability with value between 0 and 1, which is used to update labels through going back to original know label information. And *ADJ* represents the original adjacency matrix. Thus, we performed the label propagation on miRNA and disease transition matrices as:

$\left\{\begin{matrix} YM_{t+1}=(1-\alpha)\cdot TM\cdot YM_{t}+\alpha\cdot ADJ \\ YD_{t+1}=(1-\alpha)\cdot TD\cdot YD_{t}+\alpha\cdot ADJ^{T} \end{matrix}\right.$ (15)

In the adjacency matrix *ADJ* of miRNA and disease, each row represents a miRNA and each column represents a disease. *α* here is set to 0.5. The final score was calculated by:

$score=\beta\cdot YM+(1-\beta)\cdot YD^{T}$ (16)

where *β* is a parameter with value between 0 and 1 for controlling the weights of two label propagation results. And *β* here was set to 0.6.

1. Li J, Zhang S, Wan Y, Zhao Y, Shi J, Zhou Y, Cui Q: **MISIM v2.0: a web server for inferring microRNA functional similarity based on microRNA-disease associations.** *Nucleic Acids Res* 2019.

2. Wang D, Wang J, Lu M, Song F, Cui Q: **Inferring the human microRNA functional similarity and functional network based on microRNA-associated diseases.** *Bioinformatics* 2010, **26:**1644-1650.

3. Yu SP, Liang C, Xiao Q, Li GH, Ding PJ, Luo JW: **MCLPMDA: A novel method for miRNA-disease association prediction based on matrix completion and label propagation.** *J Cell Mol Med* 2019, **23:**1427-1438.

4. Chen X, Yan CC, Zhang X, You ZH, Deng L, Liu Y, Zhang Y, Dai Q: **WBSMDA: Within and Between Score for MiRNA-Disease Association prediction.** *Sci Rep* 2016, **6:**21106.

5. van Laarhoven T, Nabuurs SB, Marchiori E: **Gaussian interaction profile kernels for predicting drug-target interaction.** *Bioinformatics* 2011, **27:**3036-3043.
